# Results of German external quality assessment schemes for SARS-CoV-2 antigen detection

**DOI:** 10.1038/s41598-023-40330-2

**Published:** 2023-08-14

**Authors:** Laura Vierbaum, Nathalie Wojtalewicz, Hans-Peter Grunert, Anika Zimmermann, Annemarie Scholz, Sabine Goseberg, Patricia Kaiser, Ulf Duehring, Christian Drosten, Victor Corman, Daniela Niemeyer, Holger F. Rabenau, Martin Obermeier, Andreas Nitsche, Janine Michel, Andreas Puyskens, Jim F. Huggett, Denise M. O’Sullivan, Eloise Busby, Simon Cowen, Peter M. Vallone, Megan H. Cleveland, Samreen Falak, Andreas Kummrow, Ingo Schellenberg, Heinz Zeichhardt, Martin Kammel

**Affiliations:** 1 INSTAND E.V., Society for Promoting Quality Assurance in Medical Laboratories, Ubierstr. 20, 40223 Düsseldorf, Germany; 2 IQVD GmbH, Institut für Qualitätssicherung in der Virusdiagnostik, Berlin, Potsdamer Chaussee 80, 14129 Berlin, Germany; 3 GBD Gesellschaft für Biotechnologische Diagnostik mbH, Berlin, Potsdamer Chaussee 80, 14129 Berlin, Germany; 4 Institute of Virology, Charité - University Medicine Berlin; National Consultant Laboratory for Coronaviruses; German Centre for Infection Research, Berlin, Berlin, Germany; 5 Institute for Medical Virology, University Hospital, Goethe University Frankfurt, Frankfurt, Hessen Germany; 6 Medizinisches Infektiologiezentrum Berlin, Berlin, Germany; 7 Robert Koch Institute, Highly Pathogenic Viruses, Centre for Biological Threats and Special Pathogens, WHO Reference Laboratory for SARS-CoV-2 and WHO Collaborating Centre for Emerging Infections and Biological Threats, Robert Koch Institute, Berlin, Germany; 8 National Measurement Laboratory, LGC, Teddington, Middlesex UK; 9 School of Biosciences and Medicine, Faculty of Health and Medical Science, University of Surrey, Guildford, UK; 10 NIST, National Institute of Standards and Technology, Applied Genetics Group, Biomolecular Measurement Division, Materials Measurement Laboratory, Gaithersburg, MD USA; 11 Physikalisch-Technische Bundesanstalt, Berlin, Germany; 12 Institute of Bioanalytical Sciences, Center of Life Sciences, Anhalt University of Applied Sciences, Bernburg, Saxony-Anhalt Germany

**Keywords:** Biological techniques, Immunological techniques, Infectious diseases, Viral infection, Laboratory techniques and procedures, Diagnosis, Public health

## Abstract

The COVID-19 pandemic illustrated the important role of diagnostic tests, including lateral flow tests (LFTs), in identifying patients and their contacts to slow the spread of infections. INSTAND performed external quality assessments (EQA) for SARS-CoV-2 antigen detection with lyophilized and chemically inactivated cell culture supernatant of SARS-CoV-2 infected Vero cells. A pre-study demonstrated the suitability of the material. Participants reported qualitative and/or quantitative antigen results using either LFTs or automated immunoassays for five EQA samples per survey. 711 data sets were reported for LFT detection in three surveys in 2021. This evaluation focused on the analytical sensitivity of different LFTs and automated immunoassays. The inter-laboratory results showed at least 94% correct results for non-variant of concern (VOC) SARS-CoV-2 antigen detection for viral loads of ≥ 4.75 × 10^6^ copies/mL and SARS-CoV-2 negative samples. Up to 85% had success for a non-VOC viral load of ~ 1.60 × 10^6^ copies/mL. A viral load of ~ 1.42 × 10^7^ copies/mL of the Delta VOC was reported positive in > 96% of results. A high specificity was found with almost 100% negative SARS-CoV-2 antigen results for HCoV 229E and HCoV NL63 positive samples. Quantitative results correlated with increasing SARS-CoV-2 viral load but showed a broad scatter. This study shows promising SARS-CoV-2 antigen test performance of the participating laboratories, but further investigations with the now predominant Omicron VOC are needed.

## Introduction

Since the onset of COVID-19 in 2019, quantitative reverse transcription polymerase chain reaction (RT-qPCR) methods have been considered the gold standard for the detection of SARS-CoV-2 in infected individuals as a result of their high sensitivity and specificity^1^. However, these methods are challenging to handle as they require specialized laboratory equipment and trained professionals, have relatively long processing, and reaction times^2–5^. A lack of sufficient infrastructure means that it can take up to a week after sample collection before the test results are obtained^6–8^. Antigen-based lateral flow tests (LFTs) provide the opportunity to diagnose symptomatic individuals earlier, thereby potentially enabling interruption of virus transmission sooner^9^. In addition, antigen-LFTs could be a more cost-effective method than nucleic acid amplification techniques (NAAT)^10^, which is an important factor in low- and middle-income countries. As a result, the WHO has defined minimum performance criteria for antigen-LFTs in comparison to NAAT-based methods, and recommends performing the antigen-LFT within the first five to seven days after the onset of symptoms^9^.

A previous comparative evaluation of the sensitivity of 122 CE-marked SARS-CoV-2 antigen LFTs revealed a wide range of sensitivity for an evaluation panel consisting of pooled clinical specimens with high, medium and moderate viral loads. The study concluded that most of the SARS-CoV-2 antigen-LFTs analyzed appeared to be suitable in detecting acute infections with high viral loads; however, 26 out of the 122 LFTs indicated a lower sensitivity and some LFTs failed completely^11^. In addition, a meta-analysis conducted by Dinnes et al., which compared various published antigen-LFT study results, showed that, while many LFTs met the WHO specificity criteria, only seven were able to meet the sensitivity criterion in the pooled results of all of the analyzed studies^12^. A few LFTs were found to meet the criteria in individual studies but not in the pooled analysis. These results highlight the need for good quality control procedures to enable the best possible diagnostic performance.

The Society for Promoting Quality Assurance in Medical Laboratories e.V. (INSTAND) has been designated a German reference institution by the German Medical Association. In March 2021 INSTAND became one of the first institutions worldwide to introduce an external quality assessment (EQA) scheme, also known as a proficiency test, for SARS-CoV-2 antigen detection.

In this paper, we present the qualitative antigen results for both antigen-LFTs and automated immunoassays from the three first EQA surveys performed in 2021 as well as the quantitative results of the automated immunoassays from March 2021. This report is the first to evaluate consecutive EQA surveys for SARS-CoV-2 antigen detection.

## Materials and methods

### Sample materials—properties and preparation

Each survey consisted of five different samples. SARS-CoV-2 positive samples contained supernatants of cell cultures infected with a non-VOC SARS-CoV-2 or the SARS-CoV-2 Delta variant of concern (VOC) B.1.617.2 (Table 1). For preparation of cells and viruses see^13^. Briefly, different strains of SARS-CoV-2 and alpha coronaviruses were cultivated, and the supernatants were collected and further processed as described previously. Vero cells (ATCC CRL-1586) were used for propagation of the SARS-CoV-2 viruses, Huh-7 cells (CVCL_0336) for the propagation of alpha coronavirus hCoV 229E and LLC-MK2 cells (ATCC CCL-7) for the propagation of alpha coronavirus hCoV NL63 (Table 1). The two alpha coronaviruses hCoV229E (March 2021) and hCoV NL63 (June 2021) were used as specificity controls to get an impression, whether the detection antibodies in LFT or automated immunoassays might be sufficiently specific for coronavirus SARS-CoV-2. High test specificity is needed in terms of quick and selective isolation of SARS-CoV-2 infected individuals, as the acute respiratory symptoms of the different coronaviruses are similar. In each survey, one sample was virus negative and contained cell culture lysate from non-infected MRC-5 cells (ATCC-CCL-171). See Table 1 for sample properties and distribution in the EQA surveys.

**Table 1:** Sample properties and distribution.

Isolate/Sample material	Sample No	EQA survey	SARS-CoV-2	Cell line used for propagation	Variant of Concern (VOC)	Dilution	SARS-CoV-2 RNA load (copies/mL) dPCR Conc. ± expanded uncertainty (95% confidence interval)
SARS-CoV-2, BetaCoV/Munich/ChVir984/2020_IsolatBER, cell culture supernatant	410004	2021 March	Positive	Vero	non-VOC	1: 750	(1.65 ± 1.06) × 10^7^
	410006	2021 June					
	410009	2021 June					
	410012	2021 September					
	410013	2021 September	Positive		non-VOC	1: 2372	(4.75 ± 1.68) × 10^6^
	410001	2021 March	Positive		non-VOC	1: 7500	(1.60 ± 0.96) × 10^6^
	410007	2021 June					
	410014	2021 September					
	410005	2021 March	Positive		non-VOC	1: 75,000	(1.55 ± 1.51) × 10^5^
SARS-CoV-2, hCoV-19/Germany/SH-ChVir25702_4/2021, cell culture supernatant	410015	2021 September	Positive	Vero	Delta VOC B.1.617.2	1: 500	(1.42 ± 0.18) × 10^7^
alpha coronavirus hCoV 229E, cell culture supernatant	410003	2021 March	Negative	Huh-7	–	1: 1000	–
alpha coronavirus hCoV NL63, cell culture supernatant	410010	2021 June	Negative	LLC-MK2	–	1: 1000	–
MRC-5, cell lysate	410002	2021 March	Negative	MRC-5	–	–	–
	410008	2021 June					
	410011	2021 September					

All materials containing the SARS-CoV-2 virus were chemically inactivated with beta-propiolactone (BPL). A 10 mL aliquot of SARS-CoV-2 positive cell culture supernatant was treated with 0.05% BPL for 14 h at 4 °C. Subsequently, the BPL was hydrolyzed for 2 h at 37 °C. This treatment reduced the number of plaque-forming units (PFU) from 1.21 × 10^6^ PFU/mL to 0 PFU/mL.

The samples containing the alpha coronavirus were not inactivated. All viruses were provided by the National Coronavirus Consultant Laboratory, Charité—University Medicine Berlin, Institute of Virology, Berlin, Germany.

The samples were generated as described previously^13^. In brief, the supernatants from cell cultures were diluted using a cell culture medium (Minimal Essential Medium, PanBioTech, Aidenbach, Germany), supplemented with non-essential amino acids (PanBioTech), HEPES buffer (PanBioTech) and fetal bovine serum (PanBioTech, gamma irradiated; 15% v/v for supplemented cell culture medium). Finally, 0.5 mL of the materials were aliquoted in screw cap micro tubes (2.0 mL; Sarstedt, Nümbrecht, Germany) and lyophilized. Randomly selected vials of each of the EQA samples were analyzed for stability during the period of the EQA survey and for homogeneity according to DIN EN ISO/IEC 17043:2010-05^14^ in order to show the stability of the control materials. Prior to each of the EQA surveys in March, June and September 2021, samples were proficiency tested by up to four INSTAND expert laboratories which confirmed that the samples were fit for purpose.

### Pre-study on EQAS sample suitability

To ensure the suitability of the samples for the March 2021 EQA survey, an independent pre-study was conducted to identify the most appropriate dilution of SARS-CoV-2 positive supernatant as well as to assess the effect of lyophilization on antigen detectability. A tenfold dilution series of a supernatant of a non-VOC SARS-CoV-2 was prepared (dilution from 1:75 to 1:750,000). Half of the samples were lyophilized and half were kept in liquid form. These samples were characterized by four expert laboratories and the sample manufacturer using different SARS-CoV-2 antigen-LFTs. The LFTs of Abbott—Panbio COVID-19 Ag Rapid Test and Roche (SD Biosensor) 1—SARS-CoV-2 Rapid Ag Test were used by several laboratories. Automated immunoassays were applied by two laboratories with either the SARS-CoV-2 Ag Roche Cobas [COI] or the SARS-CoV-2 Ag-CLIA Diasorin [TCID_50_/mL]. In the case of a positive result, the expert laboratories were asked to compare the intensity of the test line with the intensity of the control line and to classify the visible difference in intensity of both lines. A positive test result was classified as either ‘test line stronger than control line’ (5), ‘test line as strong as control line’ (4), ‘test line weaker than control line’ (3), ‘test line much weaker than control line’ (2) and ‘test line faint, just visible’ (1). A negative result was defined as (0). This classification was used purely for analytical evaluation and does not in any way suggest that such an assessment could be used on a patient specimen to provide any additional clinical information. Four laboratories performed additional RT-qPCR to further characterize the samples.

### Determination of SARS-CoV-2 RNA loads by RT-dPCR

Reverse transcription digital PCR (RT-dPCR) was used to analyze the SARS-CoV-2 RNA load of all SARS-CoV-2 positive EQA samples in order to correlate the EQA antigen testing results of the selected dilution levels with the determined viral loads.

RT-dPCR was used for quantification purposes by three National Metrology Institutes (NMIs): the National Measurement Laboratory (NML at LGC, UK), the National Institute of Standards and Technology (NIST, USA) and the Physikalisch-Technische Bundesanstalt (PTB, Germany).

The lyophilized samples were reconstituted in 0.5 mL molecular biology grade water (PCR grade), extracted using the Qiagen QIAamp Viral RNA mini kit, and eluted (volumes used by each laboratory as described previously^13^). These eluates were analyzed by RT-dPCR on the Bio-Rad QX200 platform using the Bio-Rad one-step RT-digital droplet (dd)PCR supermix using the CDC N1, CDC N2^15^ and China N assay^16^. The results were analyzed by NML using R version 3.6.1 and RStudio version 1.2.5001. In Table 1, the assigned values for SARS-CoV-2 RNA load are shown for the five SARS-CoV-2 positive EQA samples.

### EQA procedure

INSTAND conducted a total of three EQA surveys: March (263 participants), July (148 participants) and September 2021 (168 participants) (see Supplementary Figure S1). Participants received five samples per EQA survey. See Table 1 for details on the properties of each EQA sample.

The samples had to be reconstituted with 0.5 mL double distilled water (sterile, pyrogen-free, PCR-grade) for 20 min at room temperature. In order to standardize the results of the three EQA surveys, the laboratories were instructed to use 100 µL of the reconstituted EQA sample for the testing regardless of the LFT or automated immunoassay used, representing the entire liquid volume contained in a standard swab at the time of the study. This volume of 100 µL was directly added to the buffer/elution solution specified by the manufacturer. Then the LFT or automated immunoassay was supposed to be performed according to the manufacturers’ instructions. The laboratories were asked to report back to INSTAND with their results via the RV-Online platform (https://rv-online.instandev.de). Multiple results for each sample, obtained by different test systems, could be entered. Furthermore, the laboratories were asked to provide detailed information on the test system(s) used for each analysis, including the test kit supplier(s) and test kit(s) (see Supplementary Table S2 online). The tests used by the laboratories for qualitative detection are summarized in Supplementary Table S3.

If EQA participants reported qualitative LFT results, they were further asked to compare the visible intensity of the test line with the visible intensity of the corresponding control line according to the criteria from the pre-study (Section "Pre-study on EQAS sample suitability"). If the same sample material was used for more than one EQA survey (see Table 1), the results from the participants were aggregated and evaluated together. This was purely for the purposes of understanding how the different dilutions of EQA material may compare between laboratories and LFTs or automated immunoassays and should not be construed as a clinically appropriate practice.

In the case of automated immunoassays, quantitative values were reported in pg/mL (ScheBo SARS-CoV-2 Antigen ELISA and Lumipulse G SARS-CoV-2 Antigen assay) or Tissue Culture Infection Dose50 (TCID50)/mL (LIAISON SARS-CoV-2 Ag assay). Only the quantitative results of the first EQA survey in March 2021 were evaluated. The quantitative results from the other surveys were not evaluated in detail due to the low number of quantitative values and thus the insufficient statistical significance.

### Data evaluation

We evaluated a total of 711 qualitative datasets from all three EQA surveys provided by 344 laboratories, as well as 36 quantitative datasets from the first survey in March provided by 36 laboratories. The evaluation was carried out in a test- and sample-dependent way (Supplementary Table S3).

Basic statistical analyses were performed using JMP 16.0 from SAS Institute (Cary, North Carolina, USA).

### Generation of images

The overlay images were generated using the GIMP–GNU Image Manipulation Program 2.10.3.

## Results

Our study comprised the validation of suitable SARS-CoV-2 antigen EQA samples and the evaluation of the interlaboratory results of the qualitative and quantitative detection of SARS-CoV-2 antigens from three EQA surveys (March, June and September 2021) with five samples each (Table 1).

### Pre-study results

**Figure 1:**
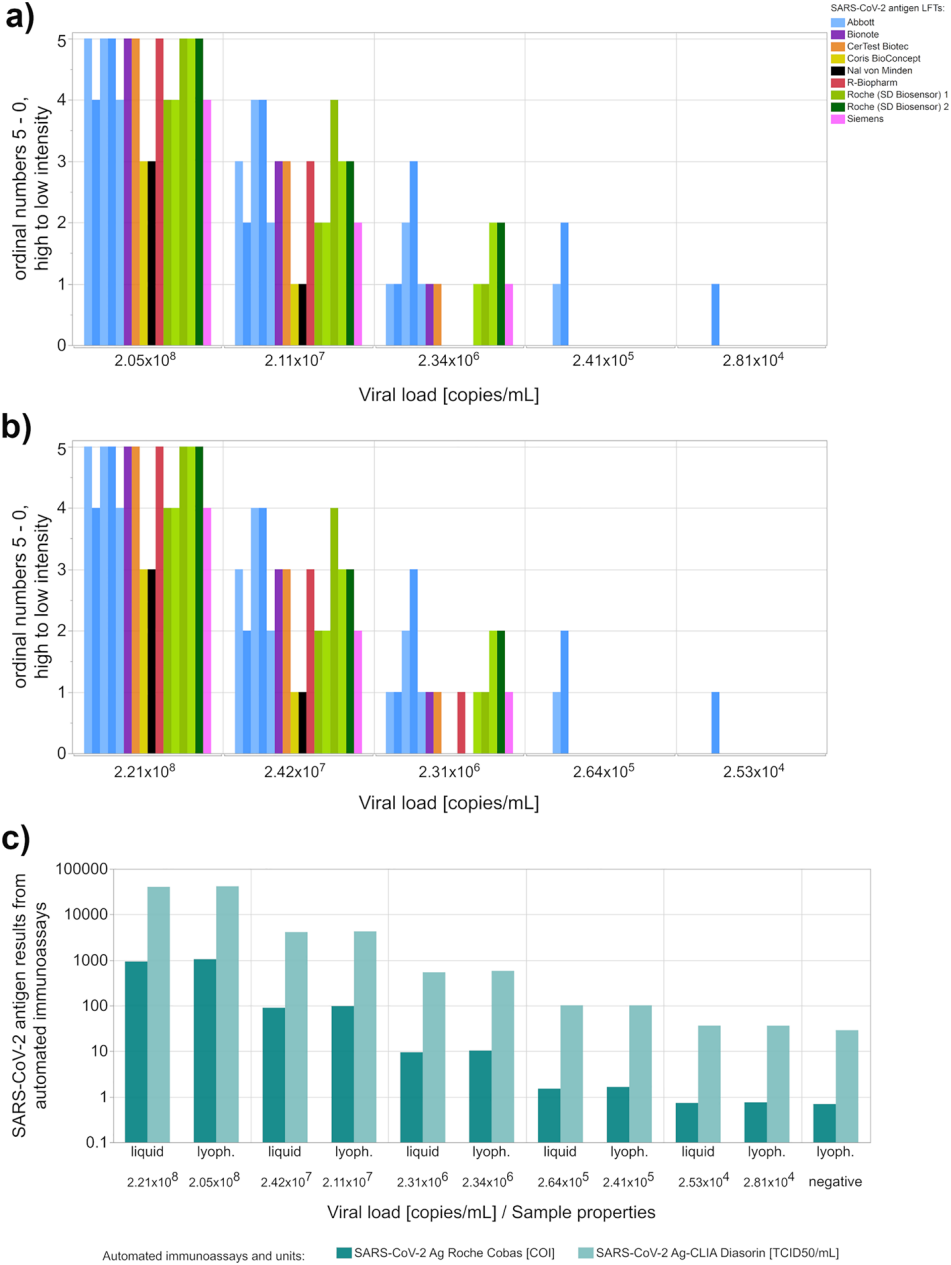
For the development and characterization of suitable EQA sample material, a dilution series of SARS-CoV-2 cell culture supernatants and a SARS-CoV-2 negative sample were measured by antigen LFTs (**a**, **b**) and automated immunoassays (**c**) by four expert laboratories and the manufacturer. Test line intensities were interpreted in comparison to the respective control line intensity and are shown for (**a**) lyophilized sample material and (**b**) for liquid sample material. Applied grading system: ‘test line stronger than control line (5), ‘test line as strong as control line’ (4), ‘test line weaker than control line’ (3), ‘test line much weaker than control line’ (2) and ‘test line faint, just visible’ (1), a negative result was defined as (0). Results from automated immunoassays are also compared for lyophilized and liquid sample material (**c**). The different tests used are shown in different colours. The LFTs of Abbott—Panbio COVID-19 Ag Rapid Test (light blue) and Roche (SD Biosensor) 1—SARS-CoV-2 Rapid Ag Test (light green) were used by several laboratories. Automated immunoassays were applied by two laboratories with either the SARS-CoV-2 Ag Roche Cobas [COI] or the SARS-CoV-2 Ag-CLIA Diasorin [TCID_50_/mL].

Since the cell culture supernatant samples where chemically inactivated their suitability for LFTs was initially assessed. The SARS-CoV-2 RNA quantity was evaluated as a surrogate for viral, and thus epitope quantity, for the positive samples used in these EQA surveys. In the pre-study described in Section "Pre-study on EQAS sample suitability", four expert laboratories and the sample manufacturer confirmed that lyophilization did not affect the detectability of the SARS-CoV-2 antigen in the LFTs and automated immunoassays. For the March EQA, a dilution of 1:750 was selected for preparation of one of the five EQA samples since, in the pre-study, mainly band intensities between ‘test line much weaker than control line’ (2) and ‘test line weaker than control line’ (3) were observed at this dilution, corresponding to a test sample that was reliably detectable without excessively high viral load. The 1:7,500 and 1:75,000 dilution levels produced lower band intensities and were used in the March 2021 EQA to test the detectability of samples that showed borderline results (Fig. 1, see Supplementary Table S1 online).

The differences of the determined SARS-CoV-2 RNA loads corresponded very well to the expected gradations of the individual dilution levels for the four non-VOC SARS-CoV-2 EQA samples.

### EQA results

**Figure 2:**
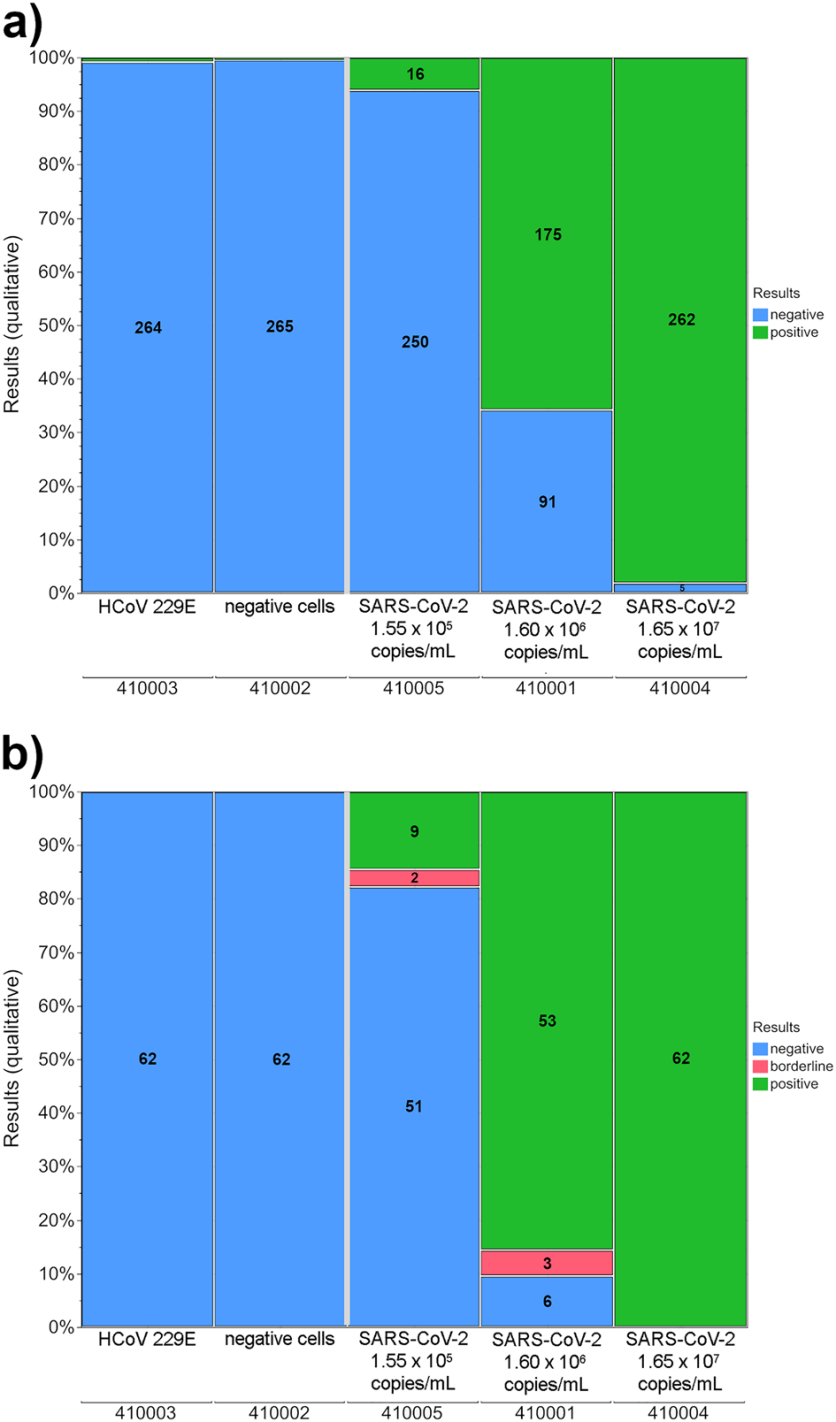
Distribution of qualitative SARS-CoV-2 antigen results for the five samples of the EQA survey in March for (**a**) lateral flow tests and (**b**) automated Immunoassays. Numbers in the columns represent the actual number of results for the corresponding category.

**Figure 3:**
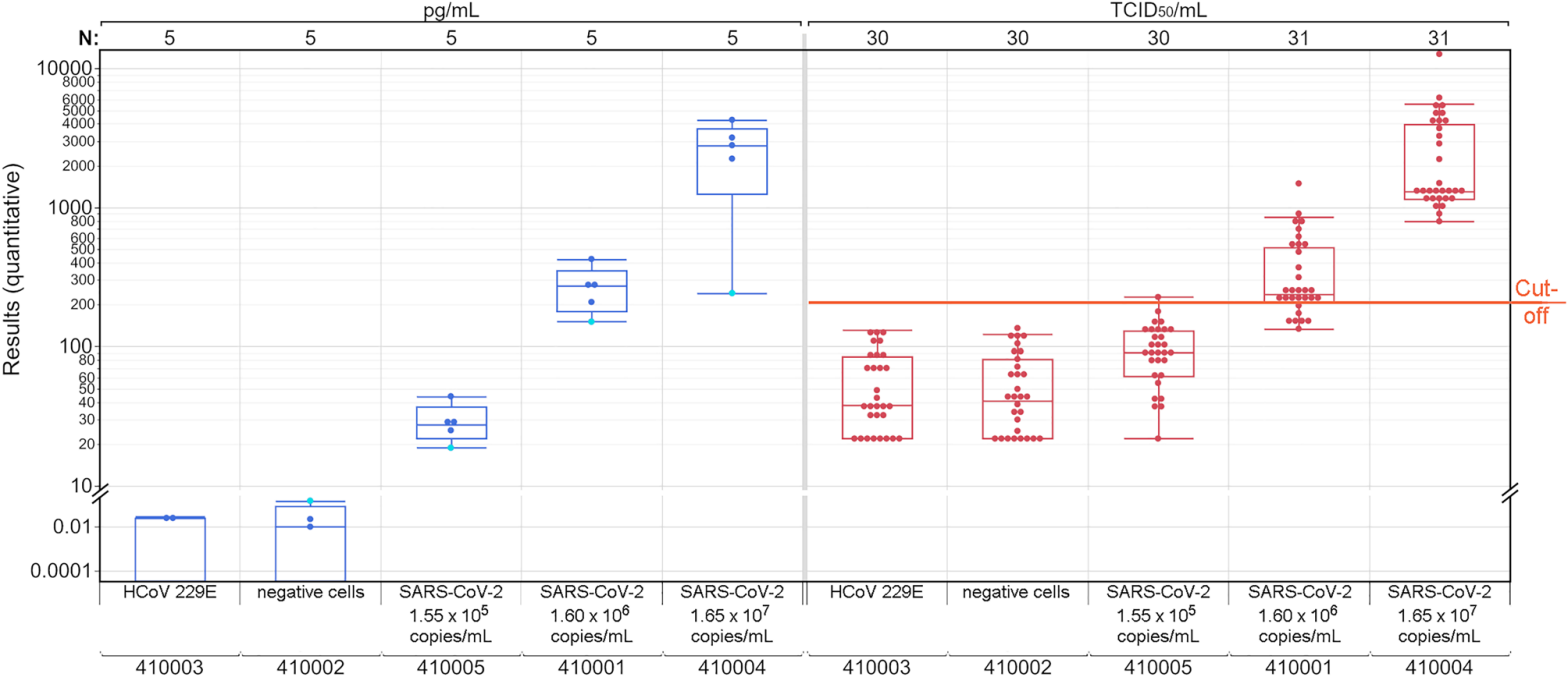
Distribution of quantitative SARS-CoV-2 antigen results for the five samples of the EQA survey in March for automated immunoassays. Participants could report their results in pg/mL (blue) or TCID50/mL (red). The light blue dot represents the ScheBo SARS-CoV-2 Antigen ELISA while the dark blue dots represent the Lumipulse G SARS-CoV-2 Antigen assay. All TCID50/mL results were obtained by the LIAISON SARS-CoV-2 Ag assay. Five results (three for HCoV 229E and two for the negative sample) indicating 0 pg/mL are not displayed in this diagram.

Since the qualitative and quantitative EQA results were similar for March, June and September, only the data for the March survey are presented graphically (Figs. 2, 3). The qualitative results for June and September can be found in the supplements (Supplementary Figures S2 and S3). The qualitative results and assay/test kit-specific results of the participants of all surveys were summarized (see Supplementary Tables S4 and S5 online).

In all three surveys, the qualitative results showed almost 100% correct results for samples with non-infected SARS-CoV-2 control cells (MRC-5 cells) as well as for samples with human alpha coronaviruses 229E and NL63, both for the LFTs (see Fig. 2a for the March EQAS and Table 2a for all three surveys) and for the automated immunoassays (see Fig. 2b for the March EQAS and Table 2a for all three surveys). In the case of the human alpha coronaviruses, used as test specificity control, 0.7% false positive results were observed for LFTs for HCoV 229E in March and 1.3% false positive results for HCoV NL63 in June (see Table 2a and Supplementary Tables S4 and S5 online). No false positive antigen results were reported for the automated immunoassays.

**Table 2:** Development of qualitative results for a) negative samples and samples containing other human corona viruses and b) a high viral load of either 4.75 × 10^6^ copies/mL or 1.42 × 10^7^ copies/mL for lateral flow tests and automated immunoassays.

(a)			Rapid Tests			Automated Immunoassays			
EQA term	Sample	Sample property	negative	positive	% negative results	negative	borderline	positive	% negative results
2021 March	410002	negative control cells (MRC-5)	265	1	99.6	62	0	0	100
2021 March	410003	HCoV 229E	264	2	99.3	62	0	0	100
2021 June	410008	negative control cells (MRC-5)	153	0	100	32	0	0	100
2021 June	410010	HCoV NL63	149	2	98.7	32	0	0	100
2021 September	410011	negative control cells (MRC-5)	159	0	100	38	0	0	100

(b)			Rapid Tests			Automated Immunoassays			
EQA term	Sample	Sample property	negative	positive	% positive results	negative	borderline	positive	% positive results
2021 March	410004	non-VOC 1.65 × 10^7^ copies/mL	5	262	98.1	0	0	62	100
2021 June	410006		3	150	98.0	0	0	32	100
	410009		2	151	98.7	0	0	32	100
2021 September	410012		4	155	97.5	0	0	38	100
2021 September	410013	non-VOC 4.75 × 10^6^ copies/mL	9	150	94.3	1	0	37	97.4
2021 March	410001	non-VOC 1.60 × 10^6^ copies/mL	91	175	65.8	6	3	53	85.5
2021 June	410007		71	82	53.6	4	3	25	78.1
2021 September	410014		71	88	55.3	11	1	26	68.4
2021 March	410005	non-VOC 1.55 × 10^5^ copies/mL	250	16	6.0	51	2	9	14.5
2021 September	410015	Delta VOC 1.42 × 10^7^ copies/mL	6	153	96.2	0	1	37	97.4

For the LFTs, samples containing the same non-VOC SARS-CoV-2 with a viral load of 1.65 × 10^7^ copies/mL were correctly identified as positive in at least 97.5% of the analyses (see Table 2b).

**Figure 4:**
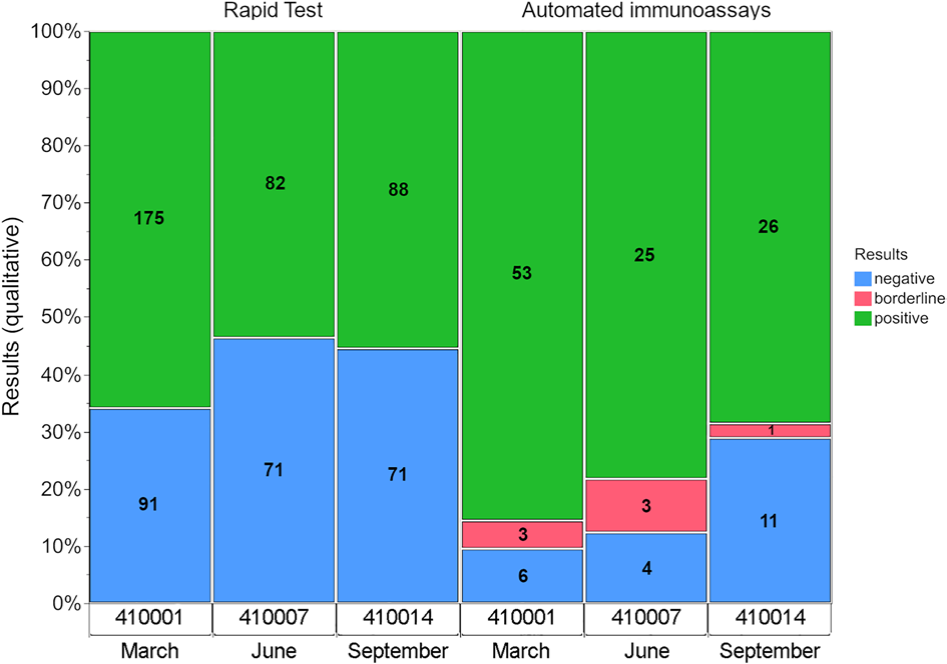
Development of the qualitative results for an estimated viral loadof 1.60 × 10^6^ copies/mL for lateral flow tests and automated immunoassays. The EQA surveys took place in March, June and September 2021.

In terms of the distribution of the qualitative results for lower viral loads of the same non-VOC SARS-CoV-2, high virus detection success rates continued to be observed down to a viral load of 4.75 × 10^6^ copies/mL (Table 2b, 94.3% positive results in September). The antigen results became divergent at a lower viral load of 1.60 × 10^6^ copies/mL. The detection rate was between 53.6 and 65.8% for the LFTs and between 68.4 and 85.5% (borderline results of 2.6% to 9.4%) for the automated systems (Table 2b and Fig. 4). The percentage of positive antigen LFT or automated immunoassay results declined distinctly for the lower SARS-CoV-2 viral load of 1.55 × 10^5^ copies/mL in March. Still, 6.0% of the LFT results and 14.5% of the automated immunoassay results were reported to be positive (Table 2b and Fig. 2).

In the case of the sample containing the SARS-CoV-2 Delta VOC with an assigned viral load of 1.42 × 10^7^ copies/mL (Table 2b), 96.2% of the results were reported to be positive for SARS-CoV-2 antigen. For the automated systems, success in SARS-CoV-2 antigen detection was nearly 100%, with only one borderline result in the case of the Delta VOC of the virus (Table 2b).

**Figure 5:**
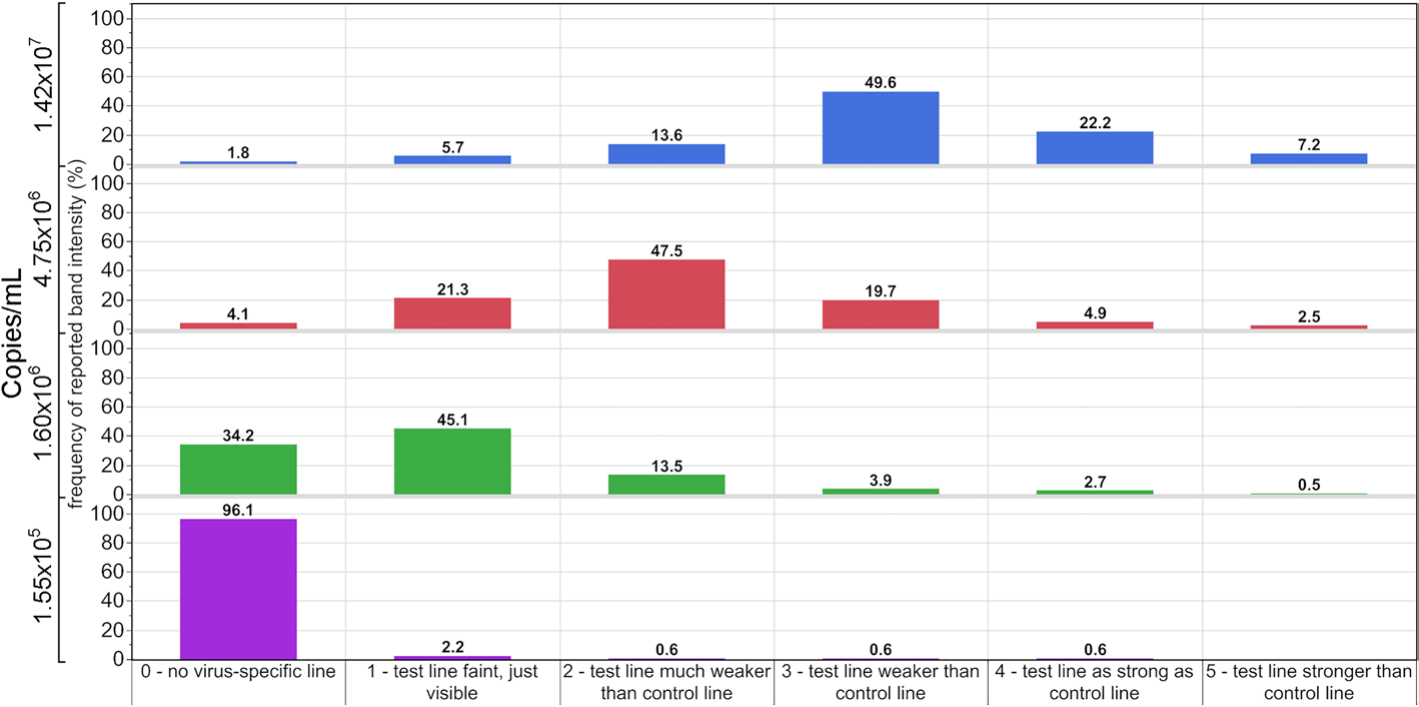
Interpretation by the participants of the line intensities compared to the intensity of the visible control line. Numbers above the columns represent the percentages for each category.

In addition to the qualitative result, between 66 and 80% of the EQA participants using LFTs compared test line intensities to the visible intensity of the control line. 49.6% of the results for the samples with the highest non-VOC SARS-CoV-2 RNA load of 1.65 × 10^7^ copies/mL showed a ‘test line weaker than control line’ (3) (Fig. 5). For non-VOC SARS-CoV-2 samples containing ~ 1.60 × 10^6^ copies/mL, 58.6% of the participants estimated weaker test line intensities of 1–2 (test line faint, just visible or test line much weaker than control line). For this sample, 34.2% of the results were found to be negative. The sample with an intermediate viral load of ~ 4.75 × 10^6^ copies/mL showed that 47.5% of the results displayed a ‘test line much weaker than the control line’ (2) (Fig. 5). For the sample with the lowest SARS-CoV-2 RNA load of ~ 1.55 × 10^5^ copies/mL, 4.0% of the participants were able to visually detect the SARS-CoV-2 antigen successfully (Fig. 5). The extent to which these values are influenced by differences in an individual’s visual ability cannot be assessed.

The distribution of the quantitative antigen results of the automated systems in March is shown in Fig. 3. The quantitative results in pg/mL or TCID_50_/mL correlated well with the estimated viral load of the samples. The value distribution of the reported TCID_50_/mL results showed an overlap between the different viral loads, for example between the samples with the lowest viral load of 1.55 × 10^5^ copies/mL and the SARS-CoV-2 negative samples (March). For the SARS-CoV-2 negative samples, which contained either non-infected MRC-5 cells or HCoV 229E, antigen concentrations were below 0.15 pg/mL or scattered around the median value of 40 TCID_50_/mL.

## Discussion

This study investigates the interlaboratory performance of SARS-CoV-2 antigen testing for both antigen-LFTs and automated immunoassays. Although the RT-qPCR methods are considered to be the gold standard for diagnosing COVID-19 through the detection of SARS-CoV-2 RNA, antigen testing provides and alternative solution that is rapid, inexpensive and—in many cases—laboratory-independent diagnostic detection of SARS-CoV-2^9,10^.

The pre-study demonstrated that the EQA materials were suitable for SARS-CoV-2 antigen testing by both LFTs and automated immunoassays for both lyophilized and liquid samples (Fig. 1). The qualitative EQA results for SARS-CoV-2 negative samples showed overall good analytical sensitivity and specificity.

All methods were able to detect the positive samples with an estimated SARS-CoV-2 viral load of ~ 1.42 × 10^7^ copies/mL (Fig. 2). LFTs had a high, 94% success rate for detecting the virus when viral load was ~ 4.75 × 10^6^ copies/mL. This success rate decreased to between 54 and 66% when there was an estimated viral load of 1.17 × 10^6^ copies/mL. For the automated systems, the analytical sensitivity was generally better, with positive rates of 68% to 86% for an estimated viral load of ~ 1.17 × 10^6^ copies/mL (Fig. 4, Table 2b). Unexpectedly, the percentage of positive results for a viral load of ~ 1.60 × 10^6^ copies/mL declined notably for the automated immunoassays. One reason for this is most likely the small sample size, which is more easily impacted by false-negative results.

So, in this study, a concentration of 1.17 × 10^6^ copies/mL seems to be the viral load, where the rapid tests begin to struggle in the detection of SARS-CoV-2.

The commercial tests used by the laboratories could not be directly ranked according to quality based on the individual results, presented in Supplementary Tables S4 and S5 online, since (i) laboratory-intrinsic influences might have predominated, (ii) the influence of swabs used in routine diagnostics was deliberately omitted and (iii) some LFTs or automated immunoassays were only used by a handful of laboratories.

For the samples used in the EQA survey in March 2021, median antigen concentrations reported for the automated immunoassays correlate well with the different estimated viral loads (Fig. 3). However, scatter in reported TCID_50_/mL antigen concentrations led to an overlap in the result distribution between the EQA samples with a tenfold difference in the estimated viral load. An overlap in the reported concentrations was also found between the SARS-CoV-2 negative sample and the sample with a SARS-CoV-2 RNA load of ~ 1.60 × 10^5^ copies/mL (Fig. 3). This indicates that the antigen concentration of this sample is close to the manufacturer’s cut off of 200 TCID_50_/mL.

Encouragingly, a high analytical specificity was found with respect to the other human coronaviruses HCoV 229E and HCoV NL63, with almost 100% negative rate for SARS-COV-2 antigen detection using both LFTs and automated immunoassays. Thus, the detection antibodies of the LFTs and immunoassays appeared to be sufficiently specific to detect SARS-CoV-2 antigens. This will be helpful for a quick and selective isolation of SARS-CoV-2 infected individuals using LFTs or automated immunoassays, since the acute respiratory symptoms of the different coronaviruses are similar. Other reports comparing different LFTs describe similar test performances^3,17^.

One possible explanation for the differences in analytical performance of the automated immunoassays and the LFTs in SARS-CoV-2 antigen detection at low viral load may be caused by the different workflows. The difference in the signal detection, generally visual and person-dependent for LFTs and using photodetectors in a qualified and validated test systems, might influence the interpretation of the results. Furthermore, LFTs often lack proper low positive control samples per lot which is particularly important when assessing weak line intensities.

In the September EQA, one sample containing the SARS-CoV-2 Delta VOC was included and showed similar results to the non-VOC SARS-CoV-2 virus. This observation is consistent with the results of other comparative studies^18,19^ which have shown that antigen assays that use cell culture samples were generally just as sensitive to other VOCs, such as the Alpha, Beta or Gamma VOC, as they were to the non-VOC SARS-CoV-2 virus. Contrary, reduced test sensitivities were described in antigen detection of the Omicron VOC^20,21^. Due to different antibody characteristics and various antigen binding sites, the sensitivity and specificity of the LFTs or automated immunoassays could be influenced by mutations in the antigen^22,23^. Thus, the sensitivity and specificity of each test has to be validated for each variant. Research is still ongoing^24^ and the introduction of an International Standard preparation is expected to produce better comparability.

Clinical parameters, like the severity or the course of disease, are essential factors for successful SARS-CoV-2 diagnostics^25–27^, but were not addressed by the EQA surveys presented here. Furthermore, preanalytical issues, including specimen type, collection, storage and transportation, are not covered in the concept of this EQA scheme, even though sample collection is another crucial aspect for SARS-CoV-2 testing^9^. One could get an indication of the influence of the sample collection by using a swab instead of a fixed sample volume pipetted for sample application. This could result in a further decrease in the concentration of the SARS-CoV-2 antigen in the LFT or automated system, which could have a negative impact on antigen detection, as also shown by a study by Puyskens et al.^28^ as well as by Cubas-Atienzar et al.^29^. To address this problem, the INSTAND EQA procedure was changed starting in November 2021 to include the use of a swab for sample collection. The respective results will be evaluated after two to three EQA surveys.

The strength of our INSTAND study was the use of well-defined EQA samples containing different viral loads of quantified SARS-CoV-2 non-VOC and Delta VOC and additional alpha coronaviruses. This enabled an assessment to be made of the general analytical performance of a broad number of laboratories worldwide which applied different antigen detection methods. The chemical inactivation of SARS-CoV-2 did not seem to have a negative effect on the analytical performance of any of the antigen LFTs presented in this study, which corresponds to the results previously reported by Zhou et al.^30^.

Our study does have some limitations: Since the EQA materials were derived from cell culture supernatants and, in contrast to routine diagnostics, no swabs were used in the analyses, the initial steps of sample preparation differ from the instructions in the test kit manuals. Furthermore, the sample origin leads to a discrepant matrix compared to patient material, so that EQA survey samples might contain different interfering components. Unfortunately, few of quantitative results for automated immunoassays were reported, which means limited statistical significance of the analysis. So more data is needed to confirm the findings of the quantitative results.

The fact that the analysis is based on EQA data, derived from artificial samples, one has to be careful with clinical conclusions on the quality and suitability of individual tests. Rather, they mirror the test-dependent performance of laboratories for a certain medical laboratory detection and thus provide an insight about the general quality of diagnostic testing. Interlaboratory assessments have therefore a key role in monitoring and promoting accurate and reliable diagnostic testing.

As virus detectability by the different LFTs and automated systems might be impaired depending on the virus variant, further analyses of antigen test performance for other SARS-CoV-2 VOCs, such as the Omicron VOC, would be of interest, especially at lower RNA loads (< 1.65 × 10^7^ copies/mL), and should be conducted as soon as possible.

To sum up, this study shows satisfactory analytical sensitivities of at least 94% for LFTs and automated immunoassays in case of non-VOC SARS-CoV-2 antigen detection for viral loads of ≥ 4.75 × 10^6^ copies/mL based on interlaboratory data. The success in non-VOC antigen detection by LFTs decreased to at least 53.4% for lower viral loads of ~ 1.60 × 10^6^ copies/mL, but the EQA results were still up to 85% positive for automated immunoassay detection. In the case of the Delta VOC the analytical sensitivity was found to be almost the same compared to this for a similar viral load of the non-VOC SARS-CoV-2, but the Delta VOC was only used in a high virus load of ~ 1.42 × 10^7^ copies/mL in one of the three EQA surveys analyzed. Analysis of HCoV 229E- and HCoV NL63-positive samples showed high specificity for antigen detection of SARS-CoV-2, with nearly 100% negative results. Thus, the EQA results for SARS-CoV-2 antigen detection by LFTs and automated immunoassays showed overall very promising performances of the participating laboratories. As reduced sensitivity of rapid antigen detection was described for the SARS-CoV-2 Omicron VOC in clinical performance^21^, further investigations of interlaboratory test data for the Omicron VOC will be of interest.

### NIST disclaimer

Points of view in this document are those of the authors and do not necessarily represent the official position or policies of the U.S. Department of Commerce. Certain commercial software, instruments, and materials are identified in order to specify experimental procedures as completely as possible. In no case does such identification imply a recommendation or endorsement by NIST, nor does it imply that any of the materials, instruments, or equipment identified are necessarily the best available for the purpose. All work has been reviewed and approved by the U. S. National Institute of Standards and Technology Research Protections Office. This study was determined to be “not human subjects research” (often referred to as research not involving human subjects) as defined in U. S. Department of Commerce Regulations, 15 CFR 27, also known as the Common Rule (45 CFR 46, Subpart A), for the Protection of Human Subjects by the NIST Human Research Protections Office and therefore not subject to oversight by the NIST Institutional Review Board.

### Supplementary Information

Supplementary Figures.

Supplementary Table S1.

Supplementary Table S2.

Supplementary Table S3.

Supplementary Table S4.

Supplementary Table S5.

## Data Availability

All data supporting the results of this article are provided in the supplementary information as raw data of the pre-study and anonymized raw data of the EQA survey.
