# Supplementary material for: Results of German external quality assessment schemes for SARS-CoV-2 antigen detection
Source: Sci Rep. 2023 Aug 14;13:13206. doi: 10.1038/s41598-023-40330-2 (PMC10425338; doi:10.1038/s41598-023-40330-2)
Supplement: Supplementary file 1 — Supplementary Figures. [file 41598_2023_40330_MOESM1_ESM.pdf]

Supplementary figures

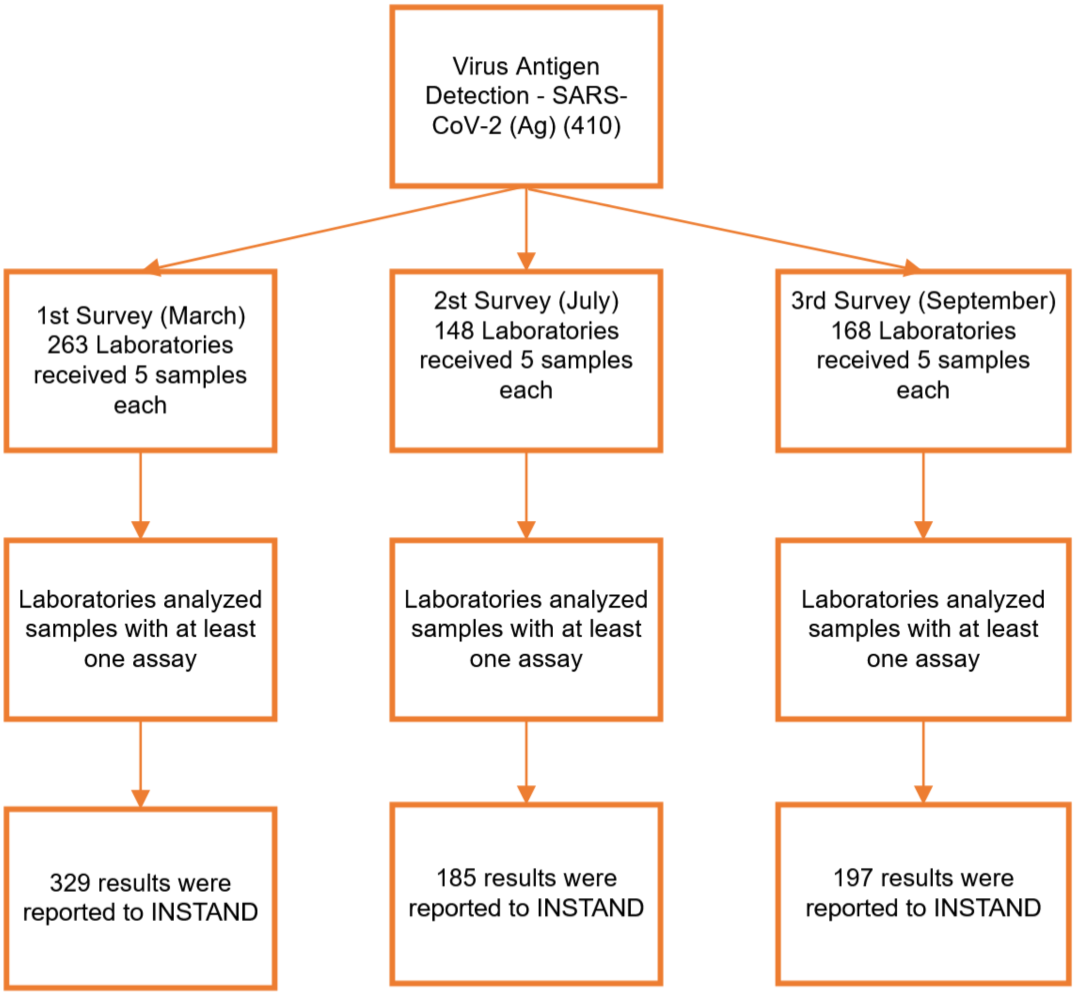

Supplementary Figure S1: Overview of the sequence of the first three EQA surveys for the detection of SARS-CoV-2 antigen conducted by INSTAND in 2021.

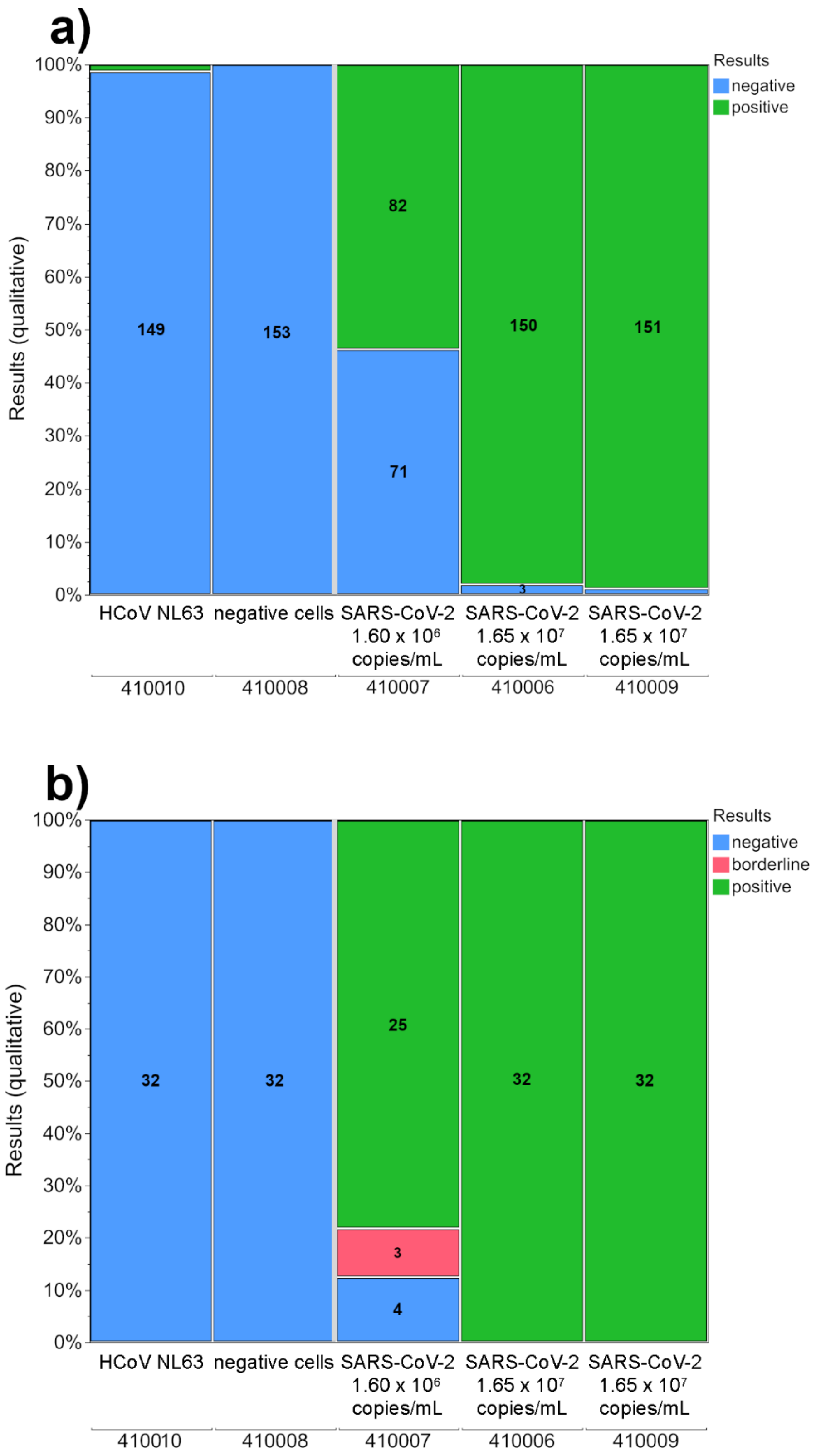

Supplementary Figure S2: Distribution of qualitative SARS-CoV-2 antigen results for the five samples of the EQA survey in June for a) lateral flow tests and b) automated immunoassays. Numbers in the columns represent the actual number of results for the corresponding category.

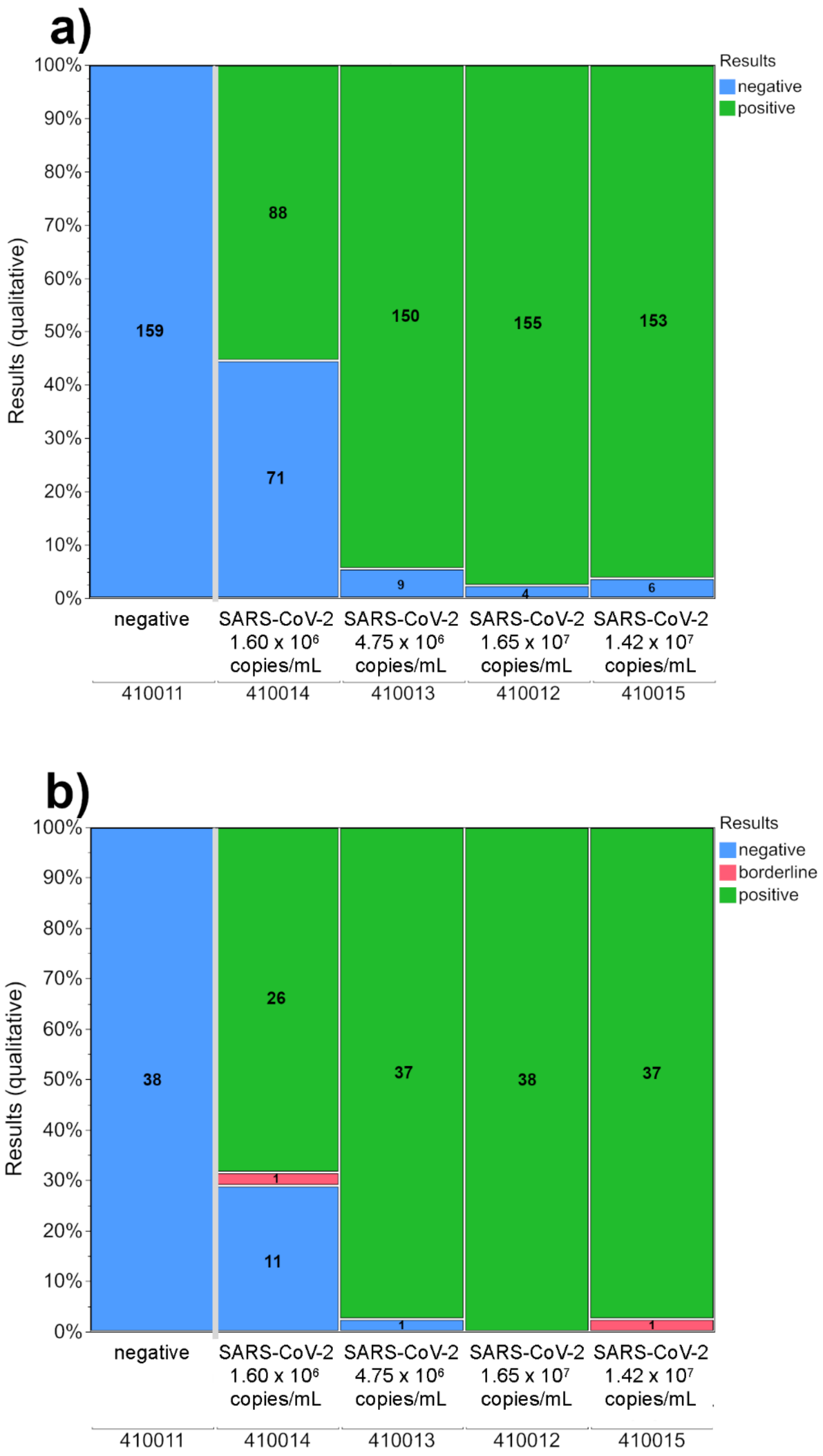

Supplementary Figure S3: Distribution of qualitative SARS-CoV-2 antigen results for the five samples of the EQA survey in September for a) lateral flow tests and b) automated immunoassays. Numbers in the columns represent the actual number of results for the corresponding category.
